# Management of Low Birth Weight in Canine and Feline Species: Breeder Profiling

**DOI:** 10.3390/ani11102953

**Published:** 2021-10-13

**Authors:** Amélie Mugnier, Sylvie Chastant, Claude Saegerman, Virginie Gaillard, Aurélien Grellet, Hanna Mila

**Affiliations:** 1 NeoCare, Université de Toulouse, ENVT, CEDEX 03, 31076 Toulouse, France; sylvie.chastant@envt.fr (S.C.); aurelien.grellet@envt.fr (A.G.); hanna.mila@envt.fr (H.M.); 2 UREAR-ULiège, FARAH, Faculté de Médecine Vétérinaire, Université de Liège, 4000 Liège, Belgium; claude.saegerman@ulg.ac.be; 3 Royal Canin, 650 Avenue de la Petite Camargue, 30470 Aimargues, France; virginie.gaillard@royalcanin.com

**Keywords:** low birth weight, dog breeder, cat breeder, management, perception, survey

## Abstract

**Simple Summary:**

Mortality rate in puppies and kittens over the first two months of age is high, estimated at around 20%. Low birth weight has been identified as a major risk factor for neonatal mortality in these species. Using an online questionnaire, we explored perceptions and management practices of breeders regarding newborns at low birth weight. Three different profiles were identified among 649 breeders. The first one included mainly dog and cat breeders who weighed newborns and monitored their suckling by the dam (controlled suckling) but did not warm them. The second group consisted of breeders of both species who did not weigh puppies or kittens to identify low birth weight or to monitor their weight afterwards. The third and final group included mostly cat breeders who routinely weighed neonates as in the first group, but who used artificial feeding rather than controlled suckling. This better knowledge of the management of puppies and kittens at high risk of neonatal mortality in the field provides the basis to establish guidelines to increase their chances of survival.

**Abstract:**

Low birth weight (LBW) has been identified as a major risk factor for neonatal mortality in many species. The aim of this survey was to determine the profiles of canine and feline breeders concerning their perceptions of, and management practices relating to, LBW individuals. An anonymous online survey was addressed to French cat and dog breeders in September 2019 via social networks. Multiple correspondence analysis and hierarchical clustering were used to explore breeders’ profiles. Three clusters were identified among the 649 breeders included in this analysis. Cluster 1 (49%) included dog and cat breeders who weighed newborns (and thus identified LBW) and controlled nursing by the dam (controlled suckling) but did not warm them up. Cluster 2 breeders (21%) of both species did not weigh puppies or kittens to identify LBW or to monitor the evolution of their weight afterwards. Cluster 3 (30%) including mostly cat breeders who weighed neonates routinely as in Cluster 1, but they practiced artificial feeding rather than controlled suckling. This survey provides a basis for better understanding of perceptions and practices regarding LBW puppies and kittens. It will be useful to provide guidelines for neonatal management to increase their chances of survival.

## 1. Introduction

Canine and feline newborns face high neonatal mortality (from birth to three weeks of age) with average live-born mortality rates close to 10% during this period [1,2,3]. Appropriate management of kittens and puppies together with the identification of at-risk newborns in order to improve survival is important for both welfare and economic standpoints.

As in other mammals (e.g., pig, sheep), low birth weight (LBW) is one of the major risk factors of neonatal mortality in puppies and kittens [4,5,6,7]. Their limited energy reserves and difficulties in suckling to obtain colostrum are, among others, the factors predisposing LBW puppies and kittens to death [8,9]. The breeder plays a pivotal role as early detection of these at-risk newborns followed by the implementation of appropriate management could reduce neonatal mortality, as demonstrated in piglets [10] and lambs [11,12]. Describing and analysing LBW management practices is the first step in reducing the high mortality rates confronting them. In production animals, survey-based studies have been conducted to provide better understanding of newborn management practices, their determinants and their consequences [13,14,15,16,17,18]. They provide a basis for evaluating current practices and targeting communication and teaching for their improvement. To our knowledge, and despite LBW being a major canine and feline health concern, the management practices and the beliefs of breeders regarding LBW have never been the subject of any scientific study.

The objective of this study was thus, for the first time, to identify canine and feline breeder profiles regarding LBW management (i.e., groups with similar management practices) based on data collected through a large online survey.

## 2. Materials and Methods

### 2.1. Survey Design 

A collaborative meeting was first organised between five veterinarians specialized in carnivore neonatology and 15 French canine and/or feline breeders to explore the management of newborns in their facilities. An online questionnaire was then drafted in French using the software Sphinx iQ 2 (Le Sphinx, Chavanod, France) and pretested by 10 people. The survey was launched in September 2019: the link was sent to dog and cat breeders belonging to the NeoCare network via Facebook and an internal mailing list (*n* = 3743 and *n* = 910, respectively). This network is made up of breeders who have voluntarily signed up to receive newsletters about the work of the team. The survey was also shared without our control. At the beginning of the questionnaire, participants were informed about the anonymity and confidentiality of the data they provide, and that by completing the questionnaire they were giving their consent for their answers to be analysed.

Among all the questions included in the survey, only those focusing on canine and feline breeders’ practices and perceptions around LBW were selected for this study. After the preprocessing of these questions (Table S1), 10 (Table 1) were used as variables in the statistical analysis. Only breeders with a response for all these 10 questions were retained for the analysis.

### 2.2. Statistical Analysis

The statistical analysis of the survey was divided into two steps in view to establishing a typology of breeders with similar practices and perceptions with regard to LBW neonates and their management. First, a multiple correspondence analysis (MCA) was performed to summarize the information contained in the selected set of categorical variables and evaluate the pattern of their relationships. MCA reduces the dimensions of these multivariate data by constructing a small number of uncorrelated synthetic factors (components or dimensions) accounting for most data variability [19]. This descriptive explanatory method also produces graphical displays making it possible to analyse the results. Only the first n dimensions (or components) that attain more than 50% of the variability explained were kept for interpretation.

For the second stage, the results obtained from MCA were used in a hierarchical cluster analysis (HCA) to place breeders into different classes. The “Euclidean” distance was calculated between the individual breeders based on the first five components selected from the MCA. Then, using Ward’s minimum variance method consolidated with the K-means method [20], HCA was used to identify homogenous clusters of breeders and their common characteristics were employed to create the profile of the cluster relating to LBW identification, management practices and perceptions. 

Statistical analyses were performed using R software, version 4.0.1 [21] and the “FactomineR” package [22].

## 3. Results

**Figure 1:**
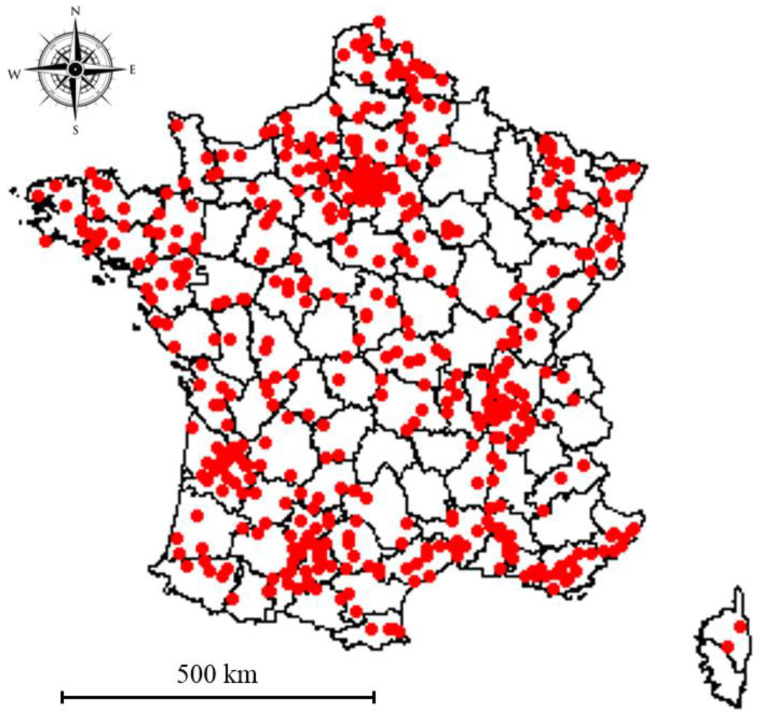
Location of the 528 French breeders who participated in the survey and whose postcode could be identified.

Six hundred and forty-nine of the 674 breeders who participated in the survey were retained for this study (answers for all the questions selected). Most of them were located in France (*n* = 514/649, 79%, Figure 1) although three other countries were represented (Belgium, *n* = 10; Switzerland, *n* = 8; Canada, *n* = 1). The country of the 116 remaining respondents (18%) could not be identified. Among the 649 participants, 48% were cat breeders, 46% were dog breeders and the remaining 6% were both dog and cat breeders. 

### 3.1. Multiple Correspondence Analysis

**Figure 2:**
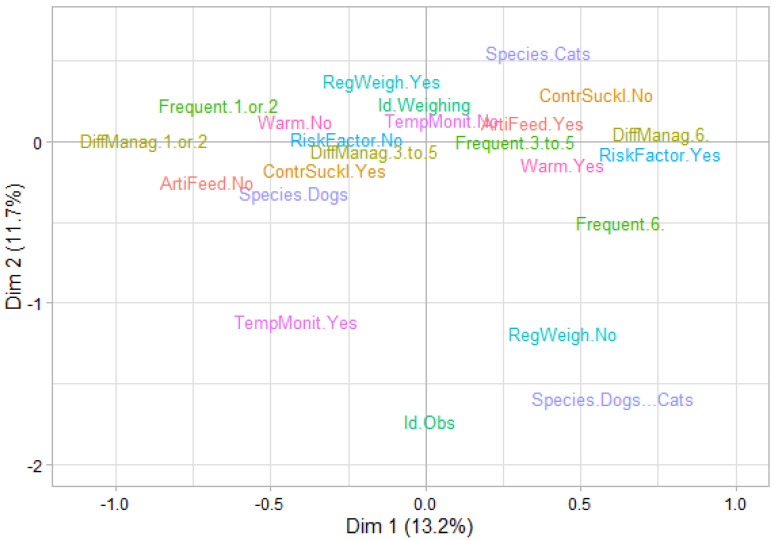
Breeders’ characteristics on factorial axes 1 and 2 according to the multiple correspondence analysis (MCA; *n* = 649 dog and cat breeders). Legend: Dim 1: dimension 1; Dim 2: dimension 2; RiskFactor.Yes/RiskFactor.No: is low birth weight (LBW) a risk factor for neonatal mortality? Yes/No; Frequent.1 or 2/Frequent.3 to 5/Frequent.6: is LBW frequent? From 1 (extremely rare) to 10 (very frequent); DiffManag.1 or 2/DiffManag.3 to 5/DiffManag.6: is LBW difficult to manage? From 1 (not at all) to 10 (very difficult); Id.Obs/Id.Weighing: which method is used to identify LBW? observation/weighing; TempMonit.Yes/Temp.Monit.No: temperature monitoring of LBW? Yes/No; ContrSuckl.Yes/ContrSuckl.No: controlled suckling for LBW? Yes/No; RegWeigh.Yes/RegWeigh.No: weight monitoring for LBW? Yes/No; Warm.Yes/Warm.No: warming for LBW? Yes/No; ArtiFeed.Yes/ArtiFeed.No: artificial feeding for LBW? Yes/No.

Five dimensions were retained for performing the MCA, accounting for a total of 52.3% of the data dispersion (i.e., variance or inertia). Figure 2 presents the cloud of modalities in the first factorial plane, i.e., with the x and y axes representing the first and the second dimensions, respectively. 

**Figure 3:**
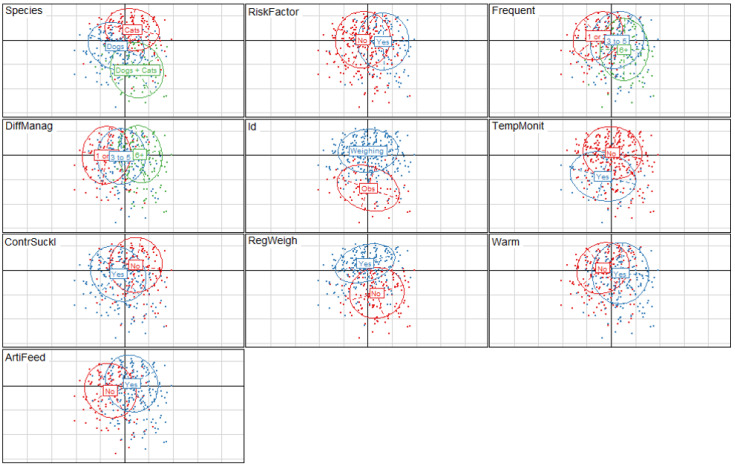
Graphical representation of multiple correspondence analysis (MCA) results based on clouds of individuals according to variables and modalities (*n* = 674 dog and cat breeders). Legend: Dim 1: dimension 1; Dim 2: dimension 2; RiskFactor: is low birth weight (LBW) a risk factor for neonatal mortality? Yes/No; Frequent: is LBW frequent? From 1 (extremely rare) to 10 (very frequent); DiffManag: is LBW difficult to manage? From 1 (not at all) to 10 (very difficult); Id: which method is used to identify LBW? observation/weighing; TempMonit: temperature monitoring of LBW? Yes/No; ContrSuckl: controlled suckling for LBW? Yes/No; RegWeigh: weight monitoring for LBW? Yes/No; Warm: warming for LBW? Yes/No; ArtiFeed: artificial feeding for LBW? Yes/No.

Figure 3 provides a graphical representation of the multiple correspondence analysis (MCA) results in the form of clouds of individuals according to variables and modalities. The first dimension explained 13.2% of the total inertia. It opposed breeders who considered LBW as a frequent issue difficult to manage and cat and mixed breeders (positive coordinates) versus breeders for whom LBW is not frequent but easy to manage and dog breeders (negative coordinates). This first axis also opposed cat breeders and mixed breeders versus dog breeders. The use of artificial feeding was also well represented on this axis. The second dimension accounted for 11.7% of the total inertia. It differentiated breeders who used weighing to identify LBW neonates and who practiced regular weighing for neonate follow-up against those who did not. The species reared on the facility was also well represented on this axis. Finally, breeders who practised controlled suckling and warming for LBW neonates were well represented on the third factorial axis, which represented 10.2% of the total inertia (Table 2). The correlation ratios (varying between 0 and 1) of the synthesis variables to the original ones, allowed identifying the most structuring variables and are presented in Table 2.

### 3.2. Typology of Breeders

**Figure 4:**
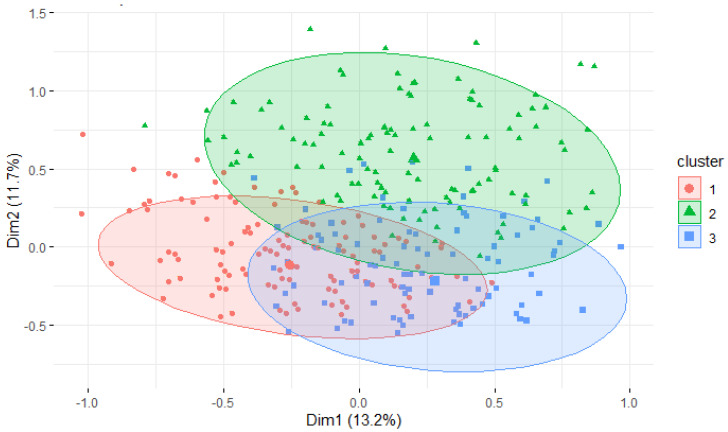
Representation of the three clusters of canine and feline breeders on the first factorial plane (*n* = 649 dog and cat breeders). Legend: Dim 1: dimension 1; Dim 2: dimension 2.

In total, three profiles of breeders regarding LBW identification, management and perception were identified and are represented in Figure 4. Detailed behaviours in each cluster are presented in Table 3.

Breeders who gave a score of 1 or 2 on the frequency of LBW and the difficulty of managing it were significantly more numerous in Cluster 1 than in the other two (Table 3). Thus, breeders in Cluster 1 considered LBW to be less frequent and less difficult to manage than in the other clusters. Moreover, they frequently used controlled suckling and weighing, but they did not often warm up the LBW neonates. 

Cluster 2 included breeders who estimated that LBW is a risk factor for neonatal mortality and who considered LBW as frequent. Moreover, the majority of breeders in this cluster did not use weighing, either at birth to identify LBW newborns or to monitor their weight evolution, were in Cluster 2. Mixed breeders were over-represented in this group.

Breeders in Cluster 3 considered LBW to be moderately frequent and difficult to manage. As in Cluster 1, they weighed LBW routinely. Among the three clusters, they practiced artificial feeding and warming most frequently, but never controlled suckling. Cat breeders were more likely to be represented in Cluster 3 (proportion significantly higher than in the two other clusters, Table 3).

## 4. Discussion

Despite the clear relationship between LBW and neonatal mortality [4,5,6,7], to the authors’ knowledge, this work is the first to explore practices and perceptions of canine and feline breeders regarding LBW neonates. Knowing current practices is the first step towards targeting communication and teaching to improve them with the final objective of reducing puppy and kitten neonatal mortality.

Data were collected from 674 breeders. This population represents less than 2% of the total number of active breeders referenced in France (according to the Société Centrale Canine and the Livre Officiel des Origines Félines, LOOF [23]). Figure 1 shows that the respondents were distributed throughout France but in a heterogeneous way with a concentration of breeders around Paris and in the south of the country. This distribution is consistent with, on the one hand, the distribution of cat breeders detailed by LOOF [23] and, on the other hand, with that of dog breeders estimated using the data of the Breeding Management System software (BMS, Royal Canin SAS, Aimargues, France; *n* = 3027 kennels; unpublished data). The respondents were recruited on a voluntary basis, mostly via Internet. Thus, a selection bias cannot be ruled out and younger breeders (more familiar with the Internet) and/or with more efficient management of LBW and/or already aware of LBW as an issue regarding neonatal mortality, were potentially more likely to respond to the survey. As this survey was written in French, the respondents were mostly French or from French-speaking countries. Further studies are needed to explore the differences in management and perceptions in other countries.

Perception regarding LBW neonates and their management varied between the different clusters (Table 3) but, surprisingly, only 25% of breeders estimated that LBW is a risk factor for neonatal mortality. There is thus a paradox considering scientific consensus on the subject [1,4,5,6]. The data reported in this study underline a lack of knowledge transfer from the scientific community to actors in the field (canine and feline breeders). Recognition of LBW as a health issue as well as its identification is indeed the first step towards improving management practices regarding these newborns [24,25] and thus improving their survival.

With regard to management practices, the current analysis revealed three clusters all of which implement actions to manage LBW (Table 3). Canine and feline neonates, and more particularly those with LBW, are born with low energy reserves and are unable to produce heat and regulate their body temperature during their first days of life [8,26]. Adequate milk intake is thus crucial, and hypothermia will occur rapidly in the case of starvation. This condition can rapidly worsen as hypothermia depresses gut motility and decreases milk digestion, leading to suckling failure and sometimes bacterial translocation from the gut to the blood stream with sepsis and death as a consequence [8,27]. As expected, and as recommended in the literature [4,7,28], weighing at birth and weight monitoring is a common practice in the kennels and catteries of this study (88% and 76%, respectively). Evaluation of weight is an easy-to-use tool, providing an immediate and objective result that allows identifying LBW neonates and controlling if weight gain is adequate. Cluster 2 included breeders who weighed significantly less, preferring to identify LBW by observation of neonate behaviour or body size. However, those breeders frequently implemented other recommended practices such as warming neonates and controlling feeding [29,30,31]. By doing so, they combat hypothermia and hypoglycemia, described as the major causes of neonatal mortality [8,27,29]. In Clusters 1 and 3, breeders preferred to ensure an adequate energy supply through controlled suckling and artificial feeding, respectively. It is interesting to note that, in Cluster 3, artificial feeding did not seem to be associated with regular temperature monitoring, contrary to the recommendations (milk formula should be provided only in newborns with body temperature >34 °C; Table 3) [30]. Lastly, canine, feline and mixed-species breeders were not equally distributed among the clusters (Table 3), suggesting that the species bred could be one of the determinants of the management practices implemented. Considering their crucial role, it could be interesting to determine the most important determinants of the management practices applied by the breeders.

Furthermore, it would be very useful to identify which practice is more or less effective in terms of managing LBW neonates, by comparing the clusters on the basis of their mortality rates. Unfortunately, the figures provided by the respondents were inconsistent and unusable for analysis, indicating that neonatal mortality is poorly estimated by canine and feline breeders; likewise for other domesticated species [32,33]. The calf mortality rate, for example, was underestimated by 20 to 50% of farmers, with 94% of them perceiving calf mortality as not being a problem [34]. The effectiveness of breeders’ management regarding LBW survival cannot be evaluated without adequate recording of neonatal mortality at a large scale. Electronic data capture systems could be very useful tools for achieving this goal. Nevertheless, it will require strong motivation from the breeder to accurately record morbidities and mortalities.

## 5. Conclusions

This survey allowed us to identify three clusters of canine and feline breeders in terms of current LBW management practices. The management practices described by breeders were complementary in the three clusters and may have a beneficial impact on LBW survival, although the relationship between those practices and the survival of newborns remains to be established. Indeed, further studies are needed to develop practical guidelines to deal with LBW (to prevent and manage them) and thus improve puppies’ and kittens’ survival. 

## Figures and Tables

**Table 1:** List of variables and their modalities used for the multivariate explanatory analysis.

Categories	Questions	Variables	Modalities	Code
	Species bred	Species	Dog	Species.Dogs
			Cat	Species.Cats
			Dog and cat	Species.Dog…Cats
Perceptions	Is LBW a risk factor for neonatal mortality?	RiskFactor	Yes	RiskFactor.Yes
			No	RiskFactor.No
	Is LBW frequent? ^1^	Frequent	1 or 2	Frequent.1 or 2
			3 to 5	Frequent.3 to 5
			6 to 10	Frequent.6
	Is LBW difficult to manage? ^2^	DiffManag	1 or 2	DiffManag.1 or 2
			3 to 5	DiffManag.3 to 5
			6 to 10	DiffManag.6
Practices	Which method is used to identify LBW?	Id	Observation	Id.Obs
			Weighing	Id.Weighing
	Temperature monitoring of LBW neonates?	TempMonit	Yes	TempMonit.Yes
			No	TempMonit.No
	Controlled suckling for LBW?	ContrSuckl	Yes	ContrSuckl.Yes
			No	ContrSuckl.No
	Weight monitoring for LBW?	RegWeigh	Yes	RegWeigh.Yes
			No	RegWeigh.No
	Warming for LBW?	Warm	Yes	Warm.Yes
			No	Warm.No
	Artificial feeding for LBW?	ArtiFeed	Yes	ArtiFeed.Yes
			No	ArtiFeed.No

^1^ From 1 (extremely rare) to 10 (very frequent); ^2^ from 1 (not at all) to 10 (very difficult). LBW: low birth weight.

**Table 2:** Correlation ratios between the variables and the five dimensions used for the MCA.

Variable	Dim 1	Dim 2	Dim 3	Dim 4	Dim 5
Artificial feeding for LBW?	0.24	0.03	0.06	0.07	0.16
Controlled suckling for LBW?	0.18	0.05	0.29	0.25	0.05
Is LBW difficult to manage?	0.40	2.5 × 10^−3^	0.20	0.26	0.23
Is LBW frequent?	0.27	0.06	0.22	0.05	0.53
Which method is used to identify LBW?	2.1 × 10^−5^	0.41	1.1 × 10^−3^	0.02	2.6 × 10^−3^
Weight monitoring for LBW?	0.06	0.45	0.01	0.02	1.8 × 10^−4^
Is LBW a risk factor for neonatal mortality?	0.19	1.6 × 10^−3^	0.02	0.22	1.8 × 10^−4^
Species bred	0.17	0.35	0.10	0.05	0.04
Temperature monitoring of LBW neonates?	0.02	0.15	0.02	0.22	0.02
Warming for LBW?	0.18	0.02	0.40	3.89 × 10^−3^	0.06

Dim: dimension. Correlation ratios were coloured according to their value: green for values higher than or equal to 0.40 (the most structuring variables for each dimension); yellow for values ranging from 0.20 to 0.40; red for values ranging from 0.10 to 0.20 (included) and white for values lower than 0.10.

**Table 3:** Distribution of the modalities in the three clusters identified through multiple correspondence and hierarchical cluster analyses with notation of significant variation.

Categories	Variables	Modalities	Cluster 1 (*n* = 318, 49%)		Cluster 2 (*n* = 138, 21%)		Cluster 3 (*n* = 193, 30%)		Total (*n* = 649)	
			Count	Proportion (CI) *	Count	Proportion (CI) *	Count	Proportion (CI) *	Count	Proportion
	Species	Cats	137	43.1% (38.4–47.8) ^a^	35	25.4% (19.4–32.2) ^b^	124	64.2% (58.2–70) ^c^	312	48
		Dogs	177	55.7% (50.9–60.3) ^a^	76	55.1% (47.7–62.3) ^a^	59	30.6% (25.1–36.5) ^b^	296	46
		Dogs + Cats	4	1.3% (0.4–2.9) ^a^	27	19.6% (14.2–26) ^b^	10	5.2% (2.8–8.6) ^c^	41	6
Perceptions	Is LBW a risk factor for neonatal mortality	No	252	79.2% (75.1–82.9) ^a^	86	62.3% (55–69.2) ^b^	150	77.7% (72.2–82.6) ^a^	488	75
		Yes	66	20.8% (17.1–24.9) ^a^	52	37.7% (30.8–45) ^b^	43	22.3% (17.4–27.8) ^a^	161	25
	Is LBW frequent	1 or 2	144	45.3% (40.6–50.1) ^a^	28	20.3% (14.8–26.7) ^b^	62	32.1% (26.6–38.1) ^c^	234	36
		3 to 5	140	44% (39.3–48.8) ^a^	66	47.8% (40.6–55.2) ^a^	101	52.3% (46.2–58.4) ^a^	307	47
		6 to 10	34	10.7% (8–14) ^a^	44	31.9% (25.3–39) ^b^	30	15.5% (11.4–20.5) ^a^	108	17
	Is LBW difficult to manage	1 or 2	85	26.7% (22.7–31.1) ^a^	23	16.7% (11.7–22.8) ^b^	35	18.1% (13.7–23.3) ^b^	143	22
		3 to 5	143	45% (40.3–49.7) ^a^	54	39.1% (32.2–46.5) ^a^	79	40.9% (35–47.1) ^a^	276	43
		6 to 10	90	28.3% (24.2–32.8) ^a^	61	44.2% (37–51.6) ^b^	79	40.9% (35–47.1) ^b^	230	35
Practices	Method used to identify LBW	Observation	15	4.7% (2.9–7.2) ^a^	57	41.3% (34.2–48.7) ^b^	6	3.1% (1.4–6) ^a^	78	12
		Weighing	303	95.3% (92.8–97.1) ^a^	81	58.7% (51.3–65.8) ^b^	187	96.9% (94–98.6) ^a^	571	88
	Weight monitoring for LBW	No	20	6.3% (4.2–9) ^a^	116	84.1% (78–89) ^b^	21	10.9% (7.4–15.3) ^a^	157	24
		Yes	298	93.7% (91–95.8) ^a^	22	15.9% (11–22) ^b^	172	89.1% (84.7–92.6) ^a^	492	76
	Artificial feeding for LBW	No	158	49.7% (44.9–54.4) ^a^	40	29% (22.7–36) ^b^	16	8.3% (5.3–12.3) ^c^	214	33
		Yes	160	50.3% (45.6–55.1) ^a^	98	71% (64–77.3) ^b^	177	91.7% (87.7–94.7) ^c^	435	67
	Controlled suckling for LBW	No	18	5.7% (3.7–8.3) ^a^	28	20.3% (14.8–26.7) ^b^	193	100% (98.5–100) ^c^	239	37
		Yes	300	94.3% (91.7–96.3) ^a^	110	79.7% (73.3–85.2) ^b^	0	0% (0–1.9) ^c^	410	63
	Temperature monitoring of LBW neonates	No	299	94% (91.4–96.1) ^a^	112	81.2% (74.8–86.4) ^b^	166	86% (81.2–89.9) ^b^	577	89
		Yes	19	6% (3.9–8.6) ^a^	26	18.8% (13.6–25.2) ^b^	27	14% (10.1–18.8) ^b^	72	11
	Warming for LBW	No	242	76.1% (71.8–80) ^a^	41	29.7% (23.3–36.8) ^b^	51	26.4% (21.2–32.2) ^b^	334	51
		Yes	76	23.9% (20–28.2) ^a^	97	70.3% (63.2–76.7) ^b^	142	73.6% (67.8–78.8) ^b^	315	49

* Letters a, b and c were used to notify significant difference between proportions of the same modality in different clusters. CI, confidence interval at 95%.

## Data Availability

Data are available from the corresponding author (A.M.) on reasonable request.

## Supplementary Materials

The following are available online at https://www.mdpi.com/article/10.3390/ani11102953/s1, Table S1: questions used in the survey addressed to cat and dog breeders translated from French into English.
