# Supplementary material for: Management of Low Birth Weight in Canine and Feline Species: Breeder Profiling
Source: Animals (Basel). 2021 Oct 13;11(10):2953. doi: 10.3390/ani11102953 (PMC8532740; doi:10.3390/ani11102953)
Supplement: Supplementary file 1 [file animals-11-02953-s001.zip › animals-1340201-supplementary.pdf]

Survey

Question in the survey	% of missing answers	Variable used for the analysis
You are: dog breeder / cat breeder / both	0.1	Dog breeder / Cat breeder / Both
Do you know the average mortality rate between birth and sale in your facility? No / Yes, precisely / Yes, approximatively	0.1	Not used
Indicate your mortality rate between birth and sale (excluding stillbirths)	30	Not used
Which conditions most influence the risk of mortality between birth and 2 months of age?<br>16 possibilities: low birth weight; presence of a malformation; low body temperature; low weight gain during the first days of life; a sick puppy/kitten; a newborn staying away from its litter; a litter of large size; a very small litter size (1 or 2); difficult farrowing; low maternal milk production; exaggerated maternal behaviour; very poorly developed maternal behaviour; the presence of a sick mother; the presence of sick puppies/kittens in the same litter; feeding/surveying during the first days of life; other (to be precised)	0.1	Low birth weight is a risk of mortality: Yes / No
Which criteria do you use to define low puppy/kitten birth weight? Weighing/BCS/Vitality/Size	0	Method to identify low birth weight: Weighing versus observation
How do you manage low birth weight puppies/kittens in your facility?<br>10 possibilities: I do nothing special; I monitor their temperature; I euthanise them; I weigh them regularly; I warm them up (incubator, hot water bottle, etc.); I control or force them to suckle their mother; I artificially feed the smallest offspring; I take them to my veterinarian for treatment; I take them to my veterinarian for euthanasia.	0	Binary variables: Artificial feeding (Yes/No) / Warming (Yes/No) / Controlled suckling (Yes/No) / Temperature monitoring (Yes/No) / Regular weighing (Yes/No)

Legend: BCS: body condition score
